# Effects of Orthodontic Treatment on Pulp Stone Formation: A Retrospective Study

**DOI:** 10.1155/2023/7381610

**Published:** 2023-04-14

**Authors:** Neda Babanouri, Sarina Sahmeddini, Marziye Rahimi Khoshmakani

**Affiliations:** ^1^Orthodontic Research Center, School of Dentistry, Shiraz University of Medical Sciences, Shiraz, Iran; ^2^Student Research Committee, Shiraz University of Medical Sciences, Shiraz, Iran

## Abstract

**Objective:**

This retrospective study was aimed at determining the incidence of dental pulp stone formation during fixed orthodontic treatment.

**Materials and Methods:**

A total of 100 patients who received fixed orthodontic treatment were included in this study. Pre- and posttreatment panoramic radiographs of the patients were examined to identify pulp stones. The data were analyzed using McNemar's and Pearson's chi-square tests to investigate the correlations between having a dental pulp stone and gender, age, treatment type, and duration.

**Results:**

Dental pulp stones were detected in 17% of patients on pretreatment panoramic radiographs and 35% of patients on posttreatment panoramic radiographs. The incidence of pulp stones sharply increased in the pre- and posttreatment radiographs (38%) (*P* < 0.001). In addition, there were associations between age, treatment duration, and the incidence of pulp stones (*P* < 0.05). Nevertheless, no associations were found between treatment type, gender, and the presence of pulp stones. Dental pulp stones were most frequently observed in first molars (62%), followed by second molars (36%).

**Conclusion:**

Fixed orthodontic treatment may trigger pulp irritation and calcification, resulting in the formation of pulp stones. Although pulp stones have no serious consequences, an orthodontist must consider the probability of pulp stone formation because it can cause difficulties in endodontic treatment.

## 1. Introduction

Pulp stones are foci of calcified masses in the dental pulp that can be attached or free in the pulpal area [1] and need to be diagnosed because they can be associated with mild to severe pulpal pain [2], leading to endodontic complications and jeopardizing the outcome of root canal treatment [3]. Moreover, since the pathogenesis of calcifications of pulpal stones is similar to calcifications in other body parts, including the cardiovascular and kidney, diagnosing pulp stones by routine dental radiographs may be useful for the early identification of potential systemic diseases [4].

One of the problems associated with pulp stones is that, although a few factors are implicated in stimulating pulp calcifications, their etiology remains unknown. Pulp degeneration and its interactions with epithelium, age, systemic diseases, bacteria, genetics, fluoride, trauma, and orthodontic movements are predisposing factors [5–7]. Orthodontic movements can cause a complex series of tissue reactions and affect periodontal and pulpal structures [8]. Changes in pulpal respiration rate and blood flow, root resorption, secondary dentin formation, and pulpal calcification may have serious consequences for pulpal health, which can be permanent and can cause pulpal necrosis [9, 10].

Another issue is that pulp stones have variable radiographic appearances, and these variations in size, shape, and location sometimes make them difficult to recognize. They can be microscopic radio-opacities or large masses that occupy the whole pulp chamber, round or ovoid, a single mass, or several pieces, and they can be found in the pulp chamber, root canal, or both areas [11, 12]. Free pulp stones are more discernable radiographically and are the most commonly diagnosed type of pulp stones [13]. Depending on their histological structure, pulp stones are calcified into true and false forms. The true form contains irregular dentin, and the false form contains degenerated pulp calcifications [14].

Generally, pulp stones can be noticed in intact, carious, restored teeth, and even in unerupted teeth [15]. The prevalence was reported to be 8-90% depending on the type of investigation, but it can be higher because some of them cannot be seen on radiographs, and histologic evaluation methods gain higher values than radiographic methods [13, 16, 17]. In addition, the prevalence and pattern were different between males and females, maxillary and mandibular arches, and different teeth, and there is controversy surrounding the rates [18, 19].

There are few studies on the relationship between orthodontic treatment and pulp calcification [9, 10]. These studies had a small sample size, only including patients in a specific age range, and the length of their orthodontic treatment was brief; however, pulp stones were occasionally observed following orthodontic treatment [20–22].

For instance, a study compared the effects of only extrusive and intrusive orthodontic forces on histological changes in the human dental pulp of 26 patients who were younger than 20 years and concluded that vacuolization and disruption of the odontoblastic layer showed statistically significant differences between teeth that underwent orthodontic forces and control teeth [20]. In another study, the effects of orthodontic force application on pulpal tissues were evaluated in 16 patients. Each had a sectional fixed appliance placed to extrude one premolar for only 14 days. The contralateral premolar was used as the control. After 14 days, both premolars were extracted, and no statistically significant difference was found in the number of fibroblasts and blood vessels. No morphological differences were observed between the control and test tissues after a 14-day period [22]. Therefore, there is no well-supported conclusion that these effects are actually caused by orthodontic movement, despite the suggestion that orthodontic forces may promote histomorphological changes in the dental pulp. This is due to the low level of evidence obtained [23]. Therefore, we attempted to research a wider group of patients with different features who received fixed orthodontic treatment to evaluate the occurrence of pulp stones after orthodontic treatment and its numerous determinants.

## 2. Method

This was a cross-sectional study of the prevalence of pulp stones in fixed orthodontic cases treated at the dental school of the Shiraz University of Medical Sciences in Iran. Before data acquisition, the Shiraz University of Medical Sciences' ethics committee granted its clearance (code IR.SUMS.DENTAL.REC.1401.104), and based on the principles of the Helsinki (ethical principles of medical research on humans), a comprehensive database of applicable records and information was recorded confidentially and with informed consent.

The inclusion criteria were all patients 12 years of age and older whose orthodontic treatment was successfully in which complete records (medical history, radiographs, clinical examination, and treatment process) were available. We collected all 120 patients' records whose treatment was completed between 2019 and August 2022. Patients with a history of trauma, uncontrolled systemic diseases, and poor-quality panoramic radiographs were excluded from this study.

Pre and posttreatment panoramic radiographs of 120 were analyzed by an experienced oral radiologist and two trained observers (interexamination). The observation was done triple within two weeks (intraexamination), and by using the same computer monitor with the same magnification of ×2, also, the viewing distance was kept constant by the observers. Pulp stones were observed as the presence of round or oval dense radiopaque structures in the pulp space, and their location and status of the involved tooth were documented from the radiographs.

Descriptive data were evaluated using means and standard deviations, and analytical data were evaluated using SPSS version 21. McNemar's test was used to compare the number of pulp stones before and after orthodontic treatments, and the Pearson chi-square test was used to determine the correlation between the presence of pulp stones and gender, age, treatment time, and duration. Statistical significance was set at *P* < 0.05.

## 3. Results

**Figure 1:**
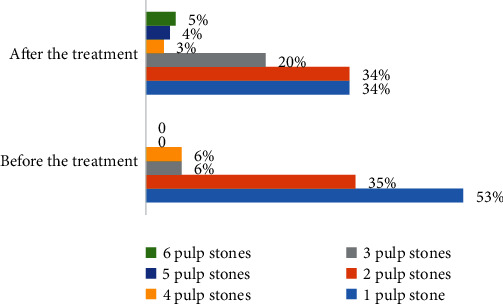
Distribution of patients with pulp stones based on the number of teeth with pulp stones.

A total of 120 patient records were evaluated; however, 20 records were excluded owing to poor-quality panoramic radiographs, withdrawal from treatment, and incomplete records. While pulp stones were observed in 17 (17%) of the patients' pretreatment panoramic radiographs, they were seen in 35 (35%) of the patients' posttreatment radiographs (Table 1).  Figure 1 presents the percentage of patients with pulp stones before and after treatment and the number of teeth that had pulp stones. Fixed orthodontic treatment significantly increased the occurrence of pulp stones (*P* < 0.0001).

Of the 100 patients, 36 had pulp stones; 27 (75%) were male, and the remaining were female. There were no significant differences in the frequency of pulp stones according to gender before and after treatment (*P* > 0.05). The mean age of these 36 patients at the beginning of the treatment was 17.22 ± 5.76. Before treatment, 12 patients were found to have newly formed pulp stones at the age of 12–18 years; this age group had the highest rate of new pulp stone formation. There was a significant association between age and the presence of pulp stones (*P* < 0.001) (Table 2).

The mean treatment duration of patients with pulp stones was 41.61 ± 12.76 months, and patients with pulp stones underwent different types of treatment. Of the 36 patients, 23 (63.9%) received nonextraction treatments, and the others underwent extractions. There was a statistically significant association between the treatment length and the frequency of pulp stones (*P* < 0.001). However, there was no correlation between the frequency of pulp stones and treatment types (Table 2).

The number of teeth with pulp stones was different in patients; some patients had only one affected tooth, and a few had up to six teeth with pulp stones (Figure 1). Of the 79 teeth with pulp stones, 33 (41.77%) teeth had multiple stones and the other had single pulp stones; 77 (97.46%) teeth had pulp stones in their pulp chamber, and two teeth had pulp stones in their root canal.  Table 3 shows the number of teeth with pulp stones in the patients; of the 79 teeth, 20 (25.31%) were restored and the others were intact.

## 4. Discussion

In this study, the incidence of pulp stones before orthodontic treatment initiation was 17%. The prevalence of pulp stones has been reported to be dissimilar among various nations due to ethnic variation [24]; however, another study in Iran asserted that 46.9% of patients had a tooth or teeth with pulp stones [18]. They used bite-wing and periapical radiographs as their observation means; however, we used panoramic radiographs, as they are more common in orthodontic treatments, and we were able to analyze both jaws simultaneously. The smaller sample size and different radiographic choices could also be reasons for this difference.

Based on these results, the frequency of pulp stones increased significantly (17% to 35%) in the radiographs taken before and after treatment. However, the relationship between orthodontic treatment and pulp stones remains controversial. These debates and differences in results might be due to the variety of fixed orthodontic appliances, treatment plans, applied forces, and tooth movements. In addition, different populations and ethnicities may be another reason for the variation in results.

Ertas et al. assessed patients who had undergone nonextraction orthodontic treatment and concluded that pulp stone prevalence sharply rose (2.2%) in the radiographs taken after the treatment [25]. Another similar study reported an overall 4% increase in cases after the commencement of orthodontic treatment [26]. Their results were similar to the current study; however, our rate of increase was higher which might be due to the fact that we did not exclude the patients who had undergone extraction orthodontic treatment. In contrast, another 2022 case-control study evaluated patients undergoing orthodontic treatment and found no discernible differences between pulp stone incidence and orthodontic treatment [27]. Moreover, Sarang did not find any significant increase in posttreatment radiographs either [28].

Han et al. concluded that severe orthodontic forces can distort pulp vessels; thus, pulp cells become anoxic. In addition, distorted vessels can reduce the pulp's ability to respond to pulpal blood impairment and maintain adequate blood supply [21]. As a result, calcifications may form around the damaged pulp tissue area. Normal pulp components are replaced by calcifications, and damaged tissue might create an inflammatory environment with unstructured, mineralized matrices that are not seen in physiological dentin. These calcifications may be linked to increased superoxide dismutase enzyme activity, and pulp stones may appear in an inflammatory setting [29].

In this study similar to several studies, there was no statistically discernible difference in the incidence of pulp stones before orthodontic treatment among genders [25–27]; in contrast, Afsari et al., in a retrospective cross-sectional study, investigated the number of pulp stones prior and following to orthodontic treatment using radiographs of patients undergoing orthodontic and nonorthodontic treatment. They found a significant relationship between gender and pulp stones because females had considerable changes in the number of pulp stones [30].

Generally, it has been reported that as age increases, the frequency of pulp stones also increases [31], whereas a study on orthodontic patients revealed no association between pulp stones and age [27]. On the other hand, our results showed a negative association between age and the frequency of pulp stones, which was in agreement with the results of Ertas et al. [25]. Hence, further investigations should be conducted to determine the relationship between age and frequency of pulp stones.

According to the literature, the mean treatment length arising from the 22 included studies considering 1089 participants was 19.9 months taking less than two years to complete comprehensive orthodontic treatment [32]. Nevertheless, the mean treatment duration of patients with pulp stones was approximately three years and a half, and we found a significant relationship between the treatment length and the frequency of pulp stones, while others did not find any correlation or did not investigate patients with prolonged treatment length [27, 30].

Moreover, we could not find any correlation between the two treatment types of extraction and non-extraction. Ramazanzadeh et al. also reported no significant relationship between tooth movement type and histological changes in dental pulp [20]. In another study on variables that differed between patients with pulp stones and controls, the authors looked into the data regarding accessory appliances used for maintenance or strength of teeth, and no significant association was found with the outcome [27]. In addition, no differences were observed between the arch and the facial sides. These findings are supported by the literature, which revealed no differences between outcomes and the facial sides or arches [25, 27, 33].

The first and second molars, respectively, exhibited pulp stones more frequently than the other posterior teeth, and the pulp stone incidence was very low in the premolars. Our results are consistent with those of numerous studies [3, 6, 19, 25, 34]. Patil et al. used Cone Beam Computed Tomography (CBCT) to determine the frequency of pulp stones in a Saudi Arabian teenage population and reported that occurrences were rare in premolars (6.68%) but significantly higher in molars (16%) [35]. Better blood supply, larger pulp chambers than other teeth, and first erupted teeth can be an attributed factor for these results to the fact that molars provide a better blood supply, which may cause calcification [6, 18], whereas Baratieri et al., using CBCT images, compared children receiving rapid maxillary expansion as the only intervention to the children receiving no orthodontic treatment to determine the impact of orthodontic force on the maxillary first molars. These findings imply that the new pulp chamber calcification was not caused by rapid maxillary expansion. In addition, because it is a multirooted tooth with a strong ability to adapt to aggression and its greater blood supply, it did not interfere with typical variations in the anchoring molars in pulp chamber dimensions [36].

Most of the teeth with pulp stones were intact in this investigation, and we did not report the decayed teeth because panoramic radiography is not accurate in detecting decays, and decayed teeth are treated before orthodontic treatment; therefore, they are not suitable for comparison. Nonetheless, another study failed to find a connection between the presence of pulp stones and the state of the tooth crown (intact, carious, and restored) [18]. In addition, the number of healthy teeth was greater than the number filled in the study by Mota et al. [27].

There have been a few studies on the relationship between orthodontic treatment and pulp calcification that had different results regarding the correlation between orthodontic treatments and pulp stones [9, 10]. Moreover, the prevalence of pulp stones is different among different populations, and there are no published articles regarding orthodontic treatments and pulp stones in an Iranian population as far as we are aware of. Therefore, the present study was aimed at investigating the relationship between pulp stones and orthodontic treatment in order to reduce the probable adverse effects of orthodontic treatment. Not considering the type of tooth movement and amount of force should be pointed out as a limitation of this study, and it would have been better if we had been able to perform vitality tests. Further studies in different populations with similar variables are needed to investigate the effects of each variable on the prevalence and incidence of pulp stones.

## 5. Conclusion

In conclusion, pulp stone formation increases after orthodontic treatment. The maximum increment was detected in the molar teeth. As a result, orthodontic treatment may change the calcification metabolism of the pulp, and orthodontic forces may promote the production of pulp stones. Therefore, clinicians should be aware of pulp stone increment and prevent it by controlling the treatment duration. Moreover, the presence of pulp stones should be assessed before the initiation of orthodontic treatment and should inform the patient of its minor effects on future endodontic treatments.

## Figures and Tables

**Table 1:** Changes in the number of patients with pulp stones before and after orthodontic treatment.

		Posttreatment		Total
		Absent	Present	
Pretreatment	Absent	64	19	83
	Present	1	16	17
Total		65	35	100

**Table 2:** Increase in pulp stones according to variables.

Variables	Pulp stones		*P* value
	Present (*n*, %)	Absent (*n*, %)	
Treatment's duration			<0.001
19-24	6 (15%)	39 (85%)	
25-30	8 (33%)	24 (67%)	
>31	21 (91%)	2 (9%)	
Age			<0.001
12-18	22 (88%)	3 (12%)	
19-24	9 (21%)	34 (79%)	
25-30	3 (11%)	23 (89%)	
31-35	1 (17%)	5 (83%)	
Gender			0.37
Male	27 (84%)	5 (16%)	
Female	8 (12%)	60 (88%)	
Treatment's type			0.55
Nonextraction	23 (32%)	48 (68%)	
Extraction	12 (41%)	17 (59%)	

^∗^ *n* = number.

**Table 3:** The frequency of pulp stones in the posterior teeth of the maxilla and mandible.

Tooth type	Maxilla		Mandible		Total
	Right	Left	Right	Left	
First premolar	0	1 (100%)	0	0	1 (1%)
Second premolar	0	0	0	1 (100%)	1 (1%)
First molar	11 (22.5%)	13 (26.5%)	12 (24.5%)	13 (26.5%)	49 (62%)
Second molar	9 (32%)	5 (18%)	7 (25%)	7 (25%)	28 (36%)
Total	39 (50%)		40 (50%)		79 (100%)

## Data Availability

The data used to support the findings of this study were supplied by the Shiraz University of Medical Sciences under license and so cannot be made freely available. Requests for access to these data should be made to Sarina Sahmeddini (sarinasahmeddini@yahoo.com).
